# ID 182 - Eladocagene Exuparvovec (Upstaza®) no Tratamento da Deficiência da Descarboxilase de L-Aminoácidos Aromáticos (AADC): alerta de monitoramento do horizonte tecnológico

**DOI:** 10.5327/2237-9622.2025.v34s1.182

**Published:** 2025-11-25

**Authors:** Mariana Del Grossi Moura, Cristiane de Cassia Bergamaschi, Jéssica Cumpian Silva, Luciane Cruz Lopes, Juliana Machado Rugolo, Viviane Del Lama Cardoso Salas, Aline do Nascimento, Silvio Barberato-Filho

**Keywords:** eladocagene exuparvovec, deficiência da enzima L-aminoácido aromático descarboxilase, monitoramento de horizonte tecnológico

## Abstract

**Introdução:**

A deficiência da enzima L-aminoácido aromático descarboxilase (AADC) é uma rara desordem neurológica genética causada por variantes patogênicas no gene da dopa descarboxilase (DDC), resultando na redução ou na ausência de atividade enzimática. A AADC é essencial para a produção de neurotransmissores monoaminas, como dopamina, serotonina, norepinefrina e epinefrina. A ausência desses neurotransmissores provoca uma série de sintomas debilitantes, como hipotonia, crises oculogíricas, distonia e atraso no desenvolvimento motor. Eladocagene exuparvovec, uma terapia genética de reposição, oferece uma nova abordagem ao tratar a causa subjacente da doença. Este estudo revisa os ensaios clínicos disponíveis sobre a eficácia e a segurança do eladocagene exuparvovec para AADC.

**Método:**

Busca sistemática foi realizada nas bases de dados Cortellis, ClinicalTrials.gov, PubMed, Embase e Cochrane Library em fevereiro de 2024. As estratégias de busca foram elaboradas utilizando os termos controlados e seus relacionados. Foram incluídos estudos clínicos que avaliaram a eficácia e a segurança do eladocagene exuparvovec para AADC, sem restrição de idioma. Os estudos foram triados com base no título, resumo e texto completo, após a remoção de duplicatas.

**Resultados:**

Quatro registros de ensaios clínicos e cinco ensaios clínicos publicados foram incluídos. Todos os registros incluídos correspondem à fase I ou II de desenvolvimento clínico e foram conduzidos nos Estados Unidos (n=1), Taiwan (n=2) e um estudo multicêntrico, envolvendo Estados Unidos, Israel e Taiwan (n=1). Dois ensaios ainda estão em andamento. Os resultados dos ensaios clínicos mostraram aumento significativo nos metabólitos de neurotransmissores (HVA e 5-HIAA) no líquido cefalorraquidiano após 3, 6 e 12 meses de terapia. Houve melhorias na função motora, evidenciadas pela pontuação nas escalas motoras aos 3, 6, 9, 12 e 18 meses após a terapia em comparação com a linha de base (PDMS-II (p<0,001), AIMS (p<0,001)). A escala de Bayley-III também revelou melhorias significativas em comparação com a linha de base nos domínios cognitivo (p=0,005) e linguagem (p=0,005). O ganho de peso corporal foi significativo no primeiro ano (p=0,005). Exames de tomografia por emissão de pósitrons (PET) mostraram aumento na observação de fluorodopa, precursor dopaminérgico, refletindo a expressão da enzima AADC. Os eventos adversos foram predominantemente leves a moderados, com um caso de hemorragia subdural que não exigiu intervenção.

**Conclusão:**

Os resultados sugerem que eladocagene exuparvovec tem potencial para restaurar a produção de dopamina e melhorar as funções motoras e cognitivas em pacientes com deficiência de AADC. Embora os dados clínicos sejam promissores, eles são baseados em amostras pequenas e ensaios de fase inicial, o que limita a generalização dos resultados. O tratamento foi bem tolerado, com eventos adversos manejáveis. Para que o medicamento seja disponibilizado no SUS, é necessário o registro pela Agência Nacional de Vigilância Sanitária (Anvisa) e a avaliação pela Conitec, considerando a eficácia, a segurança e o impacto econômico do tratamento no Brasil.

